# The Influence of the Layer Height and the Filament Color on the Dimensional Accuracy and the Tensile Strength of FDM-Printed PLA Specimens

**DOI:** 10.3390/polym15102377

**Published:** 2023-05-19

**Authors:** Doina Frunzaverde, Vasile Cojocaru, Nicoleta Bacescu, Costel-Relu Ciubotariu, Calin-Octavian Miclosina, Raul Rusalin Turiac, Gabriela Marginean

**Affiliations:** 1 Department of Engineering Science, Babes-Bolyai University, Traian Vuia Square 1-4, 320085 Resita, Romania; doina.frunzaverde@ubbcluj.ro (D.F.); nicoleta.bacescu@stud.ubbcluj.ro (N.B.); relu.ciubotariu@ubbcluj.ro (C.-R.C.); calin.miclosina@ubbcluj.ro (C.-O.M.); raul.turiac@stud.ubbcluj.ro (R.R.T.); 2 Department of Materials Science and Testing, Westphalian University of Applied Sciences Gelsenkirchen Bocholt Recklinghausen, Neidenburgerstr. 43, 45897 Gelsenkirchen, Germany; gabriela.marginean@w-hs.de

**Keywords:** fused deposition modeling (FDM), fused filament fabrication (FFF), polylactic acid (PLA), layer height, layer thickness, filament color, PLA color, dimensional accuracy, tensile strength

## Abstract

Among the FDM process variables, one of the less addressed in previous research is the filament color. Moreover, if not explicitly targeted, the filament color is usually not even mentioned. Aiming to point out if, and to what extent, the color of the PLA filaments influences the dimensional precision and the mechanical strength of FDM prints, the authors of the present research carried out experiments on tensile specimens. The variable parameters were the layer height (0.05 mm, 0.10 mm, 0.15 mm, 0.20 mm) and the material color (natural, black, red, grey). The experimental results clearly showed that the filament color is an influential factor for the dimensional accuracy as well as for the tensile strength of the FDM printed PLA parts. Moreover, the two way ANOVA test performed revealed that the strongest effect on the tensile strength was exerted by the PLA color (η^2^ = 97.3%), followed by the layer height (η^2^ = 85.5%) and the interaction between the PLA color and the layer height (η^2^ = 80.0%). Under the same printing conditions, the best dimensional accuracy was ensured by the black PLA (0.17% width deviations, respectively 5.48% height deviations), whilst the grey PLA showed the highest ultimate tensile strength values (between 57.10 MPa and 59.82 MPa).

## 1. Introduction

Additive manufacturing (AM) technologies, also named 3D printing processes, have opened a new era in many manufacturing sectors [1] and are considered to be “at the forefront of the Fourth Industrial Revolution” [2]. According to ISO/ASTM 52900:2021 [3], the term additive manufacturing includes all processes in which 3D components are made by joining materials, layer-upon-layer, following a digital model received by a computer-controlled machine (3D printer).

The growing interest in AM technologies is based on the numerous technical and economic advantages they present. The possibility of reducing the fabrication costs and shortening the production times, the ease of manufacturing parts with complex geometries, the rapid transition from the CAD model to the finished product as well as the reduction in waste have made AM technologies attractive for diverse sectors ranging from industry to education, health, and leisure [3,4,5,6,7,8].

The additive technologies market is constantly developing, reaching a volume of 12.6 billion dollars in 2020 and financial analysts predict a significant increase, “to almost triple in size between 2020 and 2026” [2]. Intensive research has been carried out regarding both the optimization of AM technologies and the development of suitable new materials for 3D and 4D printing [5,6].

Among the seven AM process categories defined by [3], one of the most commonly applied, given the relatively low cost of equipment and materials as well as the simplicity of the process, which makes it accessible even to home users, is 3D printing by material extrusion, known as FFF (fused filament fabrication) or respectively the FDM (fused deposition modeling) method [9,10]. The extruded material is a molten thermoplastic filament that is deposited on the build platform of the printer in successive layers, each of them realized of adjacent filament roads by means of a printing head. Regarding the materials suitable for FDM printing, polylactic acid, known as PLA, is considered to be the most widely used 3D filament. Its low melting point, the ease of printability even on home user machines and its low toxicity compared to other materials such as ABS are counted among the main advantages [11]. PLA is a semi-crystalline and biodegradable polymer made from renewable resources that is available in a wide range of colors.

Nowadays, AM technologies are no longer used only for rapid prototyping, but increasingly for rapid manufacturing. When it comes to the printing of end user parts by FDM, the selection of the numerous interdependent process parameters, which influence both the part quality (with respect to dimensional precision, distortions, mass deviations, porosity, surface texture, etc.), and the product’s final characteristics have proven to be a difficult problem to solve. Moreover, other than in the case of traditional manufacturing technologies, one has to consider “the strength of the part” instead of “the strength of the part material” [12], as the properties of the products obtained by FFF are strongly dependent on their mesostructure.

The topic of process optimization, aiming to obtain by FDM parts with predictable end-user properties as well as good surface quality, dimensional, form and mass accuracy, has been addressed by numerous researchers. In this respect, the influence of the following variables has been studied by experimental investigations as well as by statistical methods [13,14]: printing temperature, build plate temperature, layer thickness, printing speed, build orientation, raster angle, infill pattern and density, storage conditions before and after the printing process, post-process treatments, and aging. Less considered influential variables are the filament (material, color, diameter, producer) and the 3D printing equipment (producer, open or closed workspace), and related details are mostly not even provided among the experimental conditions when addressing other process parameters.

The layer height (or layer thickness) is one of the most approached FDM process parameters. The variation range of this 3D printing variable is set in relation to the nozzle diameter, as its maximum value can usually reach up to half of this characteristic. The layer thickness has a significant influence on the printing duration. For shorter production times and enhanced process productivity, higher values for layer thickness have to be selected, but the latter compromises the part resolution [13].

Regarding the influence of this printing parameter on the mechanical properties, most researchers agree that the strength of the PLA prints is influenced by the layer height. On the other hand, while some of them consider that this influence is low [15,16,17,18,19,20,21,22], others highlight that this influence is significant [23], or have even stated that the layer thickness is the most important factor that governs the values of the ultimate tensile strength (UTS) [24].

Neither in terms of the variation trend could there be found agreement in the previous research analyzed. In this regard, whilst most of the researchers found that the UTS of the parts manufactured by FFF is increasing with the decrease in the layer height [16,17,18,19,20,22,23,24,25,26,27,28,29,30,31,32,33,34], others concluded the opposite [14,35,36] or defined optimal values of this variable, situated inside a variation interval that was set for the optimization of the selected printing parameters [37,38,39,40,41]. These different or even controversial opinions could be explained by the fact that the chosen combinations of process parameters determined completely different thermal printing conditions as well as the distinct values adopted for the layer thickness, situated within a wide range (0.06 mm–0.6 mm) [13].

Regarding the effect of the filament type, especially its color on the mechanical properties of PLA prints realized by FDM with different layer heights, there is a lack of information. If the behavior of different colored PLA filaments is not directly addressed by the authors, the information about the color of the analyzed samples is not even mentioned in the majority of studies, showing that this factor is usually not considered to be a significant influential parameter for the properties of the FDM prints. Furthermore, filament manufacturers provide the same information for PLA filaments irrespective of their colors, thus one must consider that by adding coloring filler materials, the thermal behavior of the PLA composites might be influenced [42,43], and thereby the adhesion between adjacent roads and successive layers under the same process conditions, affecting the mechanical properties of the prints [43,44,45,46]. This effect, for PLA samples of different colors obtained under the same printing conditions, was found to be considerable, ranging up to a maximum of 29% difference in ultimate tensile strength between various colors [43], or even up to 31% [44]. No study addressing the combined influence of the color and the layer thickness on the UTS values of the PLA samples was found in the literature analyzed.

The impact of the layer height on the dimensional precision of PLA parts printed by FDM has been extensively analyzed in previous research [8]. Moreover, in the majority of cases, this process parameter is considered to have a significant influence on the print quality [8,10,47,48,49] or even to be crucial [50,51]. For layer heights varying from 0.05 mm up to 0.5 mm, most of the researchers concluded that reduced layer heights ensured better dimensional accuracy [8,50,51,52,53,54], independent of the combinations of other process parameters and that the deviations resulted along the X, Y, and Z-directions differed, depending mainly on the build orientation [8,10,50,52,54,55]. An interesting result was achieved by authors analyzing the combined effect of printing speed (50 mm/s, 60 mm/s, and 70 mm/s, respectively) and layer height, set at 0.10 mm, 0.15 mm and 0.20 mm, on the dimensional precision of a test specimen with protrusions and internal features [47]. It was pointed out that the printing speeds and layer heights have to be selected in accordance with the part geometry. However, it should be noted that the layer thicknesses were considered only in the range from 0.10–0.20 mm in that study, respectively, having values that are accepted as preferable for the dimensional accuracy of the PLA prints [51].

Regarding the influence of the layer height on the dimensional precision in connection with the filament color, very few information could be found. In this respect, only in two studies was the color of the PLA explicitly considered to be an influential variable for the dimensional accuracy, but none of them pointed out the combined effect of the filament color and layer height. Thus, the authors of [55] set the filament color at three values (white, grey, and black) and defined the optimum combination of filament color-build orientation for the best dimensional accuracy. The filament color–layer thickness–print orientation–raster angle optimization was realized only for the white PLA. In [10], four different colors (pink, grey, green, and transparent) were used to study the influence of pigmentation on the dimensional deviations, but the value of the layer thickness was the same for all samples, respectively 0.5 mm, and only the printing temperature was set at five levels. Otherwise, in studies where the filament color was not considered as a variable, complete information about the filament type selected for the experiments has been rarely provided.

Considering the above, the authors of the present study aimed to evaluate if, and to what extent, the PLA color influenced the dependences of the dimensional accuracy and the mechanical strength on the layer height in the case of samples printed by FDM. In order to point out the effect of the filament pigmentation on the previously mentioned dependences, the only process variables were the filament color (natural, black, red, and grey) and the layer height (0.05 mm, 0.10 mm, 0.15 mm, and 0.20 mm). All other process parameters were the same for all samples.

## 2. Materials and Methods

The experiments were carried out on ISO 527-2 type 1A [56] specimens with a thickness of 3 mm.

The CAD geometry of the specimen was modeled with SolidWorks (Dassault Systèmes, Vélizy-Villacoublay, France) and the process parameters were defined in the Ultimaker Cura 5.0.0 slicer (Ultimaker B.V., Utrecht, The Netherlands). An Ultimaker 2+Connect equipment (Ultimaker B.V., Utrecht, The Netherlands) with closed space was used for printing.

The printing parameters are listed in Table 1. The only variables were the layer thickness and the material color. Before printing, the 2.85 mm diameter PLA filaments, produced by Verbatim (CMC Magnetics Corporation, Taipei, Taiwan), were properly preserved in a closed space, providing protection against humidity and UV irradiation. For each material color, 20 specimens were printed (five specimens for each layer thickness), resulting in a total of 80 specimens. All specimens were printed individually (one specimen per build plate) by using the YX orientation, according to ISO/ASTM 52921:2013 [57]. No pre-process (on filament) or post-process (on printed specimens) treatments were applied.

**Figure 1:**
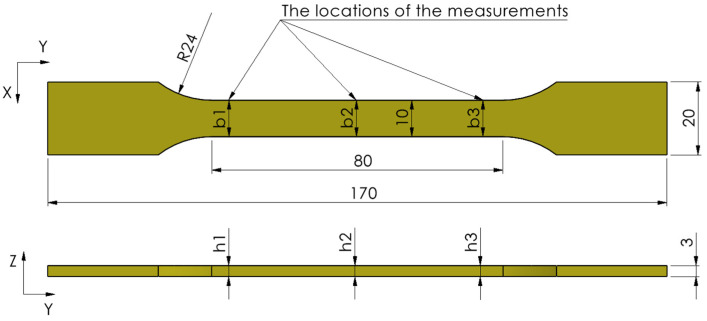
The CAD geometry of the printed tensile samples (YX-build orientation) with the positions of the dimensional accuracy measurements (b_1_, b_2_, b_3_—width measurements; h_1_, h_2_, h_3_—thickness measurements).

The assessment of dimensional accuracy was carried out by measuring the width and thickness of each specimen at three positions along the calibrated area (Figure 1). The measurements were accomplished with a Mitutoyo 293-240-30 digital micrometer (Mitutoyo Romania SRL, Otopeni, Romania), with a 0–25 mm measuring range and an accuracy of ±0.001 mm. The dimensional deviations (%) for the widths b_1_, b_2_, and b_3_ and the thicknesses h_1_, h_2_, and h_3_ were determined for each specimen. Subsequently, the average deviations of width and thickness were calculated and plotted. In addition, the mean of the cross-sectional areas of each group of specimens was calculated.

A Mecmesin Multitest 2.5 dV machine (PPT Group UK Ltd., Slinfold, UK) was used to carry out the tensile tests, according to ISO 527-1 [58] and ISO 527-2 [56]. The test speed was set at 10 mm/min. For each combination of layer height and PLA color, five tensile specimens were tested and the mean value of the ultimate tensile strengths (UTS) and the standard deviations (according to ISO 2602:1980 [59]) were calculated. The confidence level for the mean was 95%.

The mesostructure of the specimens was examined in the fractured surface and on the surface of the top layers at a magnification of 12.5× using a Leica MZ 7.5 stereomicroscope (Leica Microsystems, Wetzlar, Germany).

## 3. Results and Discussion

The experimental results are presented and interpreted in the following subsections. Aiming to facilitate the association between the PLA colors and the corresponding values measured for the dimensional deviations and the UTS, in all graphic representations, the following color palette was used: yellow for natural PLA, dark grey for black PLA, red for red PLA, and light grey for grey PLA.

### 3.1. Dimensional Accuracy

**Figure 2:**
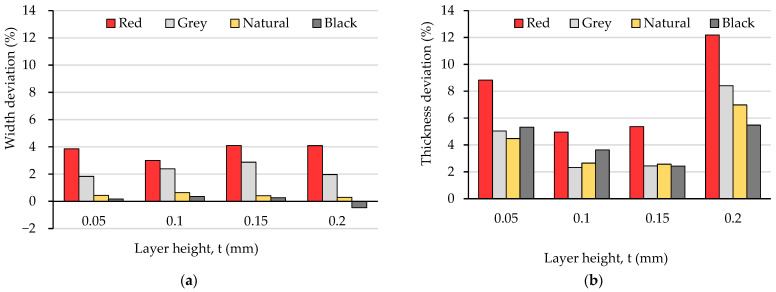
Dimensional deviations of the test specimens printed with different layer heights and filament colors: (**a**) with deviations (nominal value: 10 mm); (**b**) thickness deviations (nominal value: 3 mm).

In order to point out the influence of the layer height on the dimensional accuracy as well as the possible differences determined by the filler materials of the colored PLA filaments, the dimensional deviations of the width and the thickness in the calibrated region of the test specimens were calculated and represented in Figure 2.

Analyzing the dimensional deviations along the two considered directions, one can observe that the deviations of the specimens’ widths (Figure 2a), ranging between 0.17% (black PLA, t = 0.05 mm) and 4.10% (red PLA, t = 0.15 mm), were significantly smaller than those of the thicknesses (Figure 2b), where the minimum value was 2.32% (grey PLA, t = 0.10 mm) and the maximum one was 12.19% (red PLA, t = 0.20 mm). In agreement with [55], this phenomenon may be explained by the fact that the samples could expand freely in the vertical direction to the build plate (along the *Z*-axis, thickness), whilst the increase in the width (along the *X*-axis) was restricted by the two wall lines that made up the contour (or shell) of the prints.

**Figure 3:**
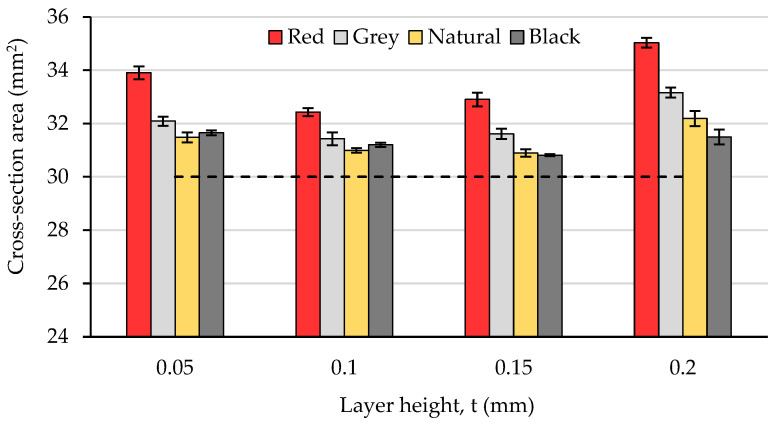
The variation in the cross-section area (theoretical area: 30 mm^2^) with the layer height and filament color.

As significant differences were found regarding both the magnitude and the variation profiles of the specimens’ width and thickness deviations with the layer height and the filament color, in order to evaluate and visualize the overall effect of the two variables on the dimensional precision, the mean value of the cross-section area was calculated and is represented in Figure 3.

The experimental results showed that for all colors and layer heights, the dimensions of the prints exceeded those of the CAD model (see the dashed line), a phenomenon that characterizes, in general, the FFF fabrication process [37].

Regarding the influence of the filament color on the dimensional precision of the samples obtained for different values of the layer height, the experimental results visualized in Figure 2 and Figure 3 obviously show that the presence of coloring additives should not be neglected in relation to the print quality. Under the same printing conditions, the best overall dimensional accuracy was ensured by the black PLA and the worst by the red PLA. The differences in the cross-sections of the red and black colored prints ranged from 3.94% for t = 0.10 mm to 11.23% in the case of t = 0.20 mm. Previous studies have indicated that the critical temperatures for glass transition and the degradation of colored polymers are influenced by the addition of coloring additives [60], so these differences may be explained by the different thermal properties of the four types of filaments.

**Figure 4:**
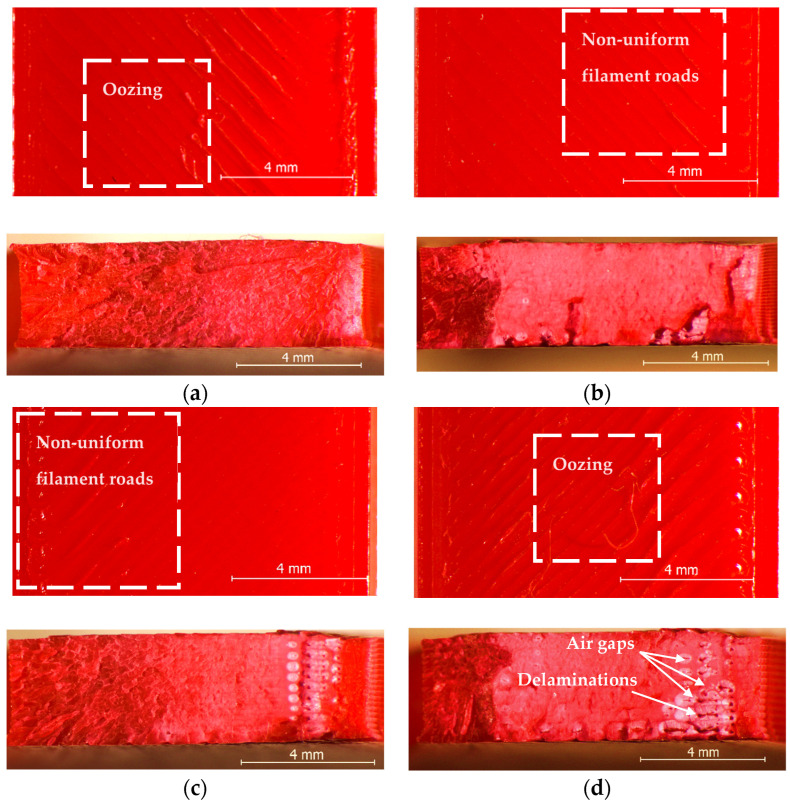
Top view (**up**) and fractured surface (**down**) of the red PLA tensile specimens printed at: (**a**) t = 0.05 mm, (**b**) t = 0.1 mm, (**c**) t = 0.15 mm, (**d**) t = 0.2 mm.

**Figure 5:**
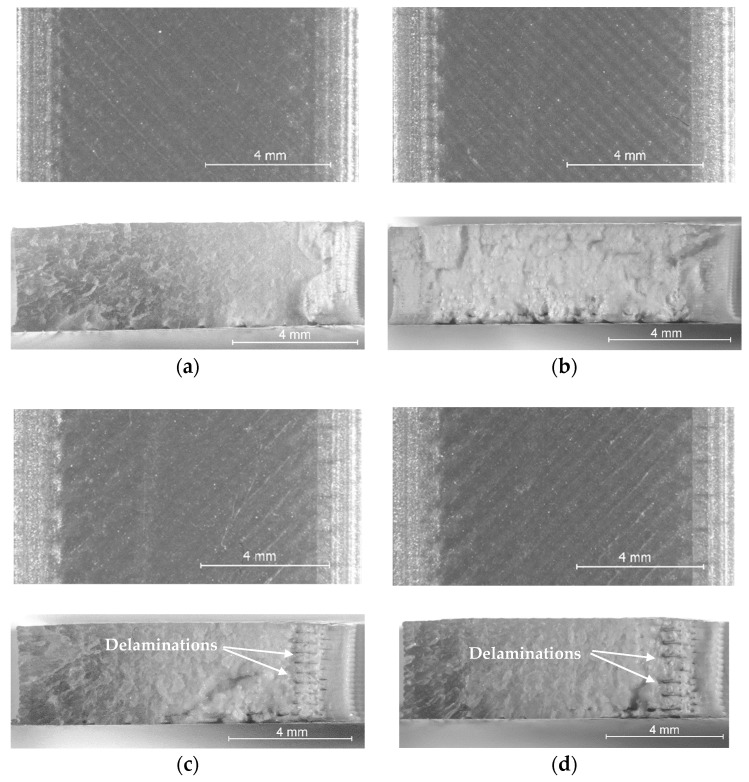
Top view (**up**) and fractured surface (**down**) of the grey PLA tensile specimens printed at: (**a**) t = 0.05 mm, (**b**) t = 0.1 mm, (**c**) t = 0.15 mm, (**d**) t = 0.2 mm.

**Figure 6:**
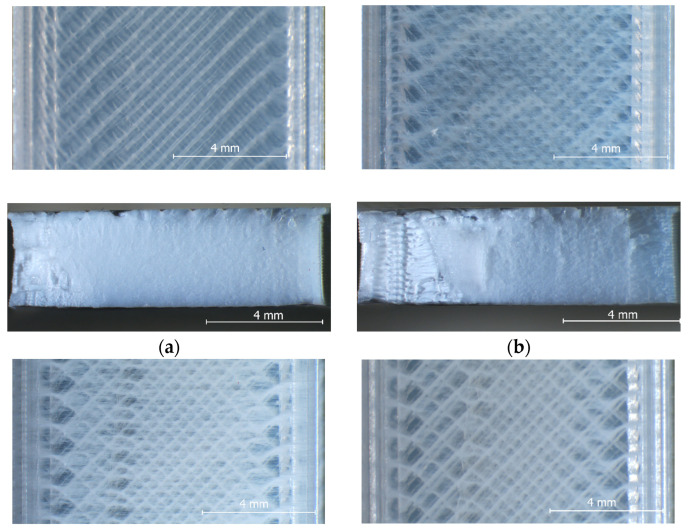

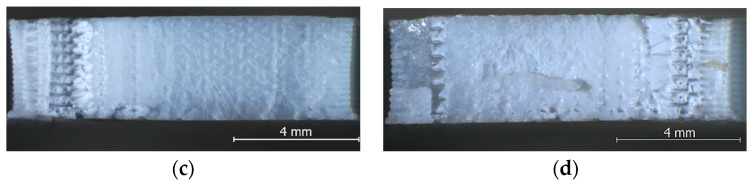
Top view (**up**) and fractured surface (**down**) of the natural PLA tensile specimens printed at: (**a**) t = 0.05 mm, (**b**) t = 0.1 mm, (**c**) t = 0.15 mm, (**d**) t = 0.2 mm.

**Figure 7:**
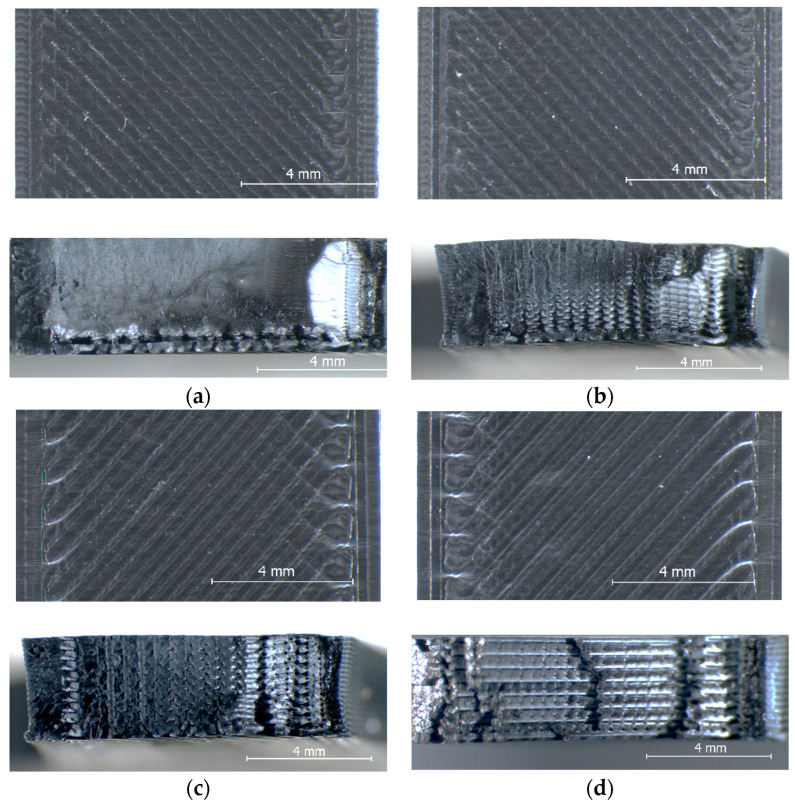
Top view (**up**) and fractured surface (**down**) of the black PLA tensile specimens printed at: (**a**) t = 0.05 mm, (**b**) t = 0.1 mm, (**c**) t = 0.15 mm, (**d**) t = 0.2 mm.

On the other hand, although the magnitudes of the samples’ dimensional deviations were obviously color-dependent, their variation trends with increasing layer thicknesses in the range from 0.05 mm to 0.20 mm were very similar. Thus, for all four PLA filament types, the best dimensional accuracy was obtained for layer heights situated between 0.10 mm and 0.15 mm. Aiming to explain these results, after tensile testing, the specimens were examined with the stereomicroscope, as described in Section 2. Representative images for the different color–layer height combinations are shown in Figure 4, Figure 5, Figure 6 and Figure 7.

As one can observe in Figure 4a, Figure 5a, Figure 6a and Figure 7a, the mesostructure of the specimens printed with the smallest layer height (t = 0.05 mm) was extremely dense for all PLA colors, indicating that during the printing process, the temperature of the already deposited layers was maintained higher, as in all other cases. On the other hand, printing with smaller layer heights involves an increased number of heating and cooling processes during the filament deposition, and hereby the formation of internal stresses and subsequent part distortions [61,62], which could explain the higher values measured for the dimensional deviations in the case of the 0.05 mm layer thickness, as also considered by the authors of [63].

The worst dimensional accuracy was obtained, for all types of PLA filaments, in the case of the 0.20 mm layer height. Figure 4, Figure 5, Figure 6 and Figure 7 reveal that the number of voids (delamination of successive layers and air gaps between adjacent roads, as marked, for example, in Figure 4d and Figure 5d increased with the increase in the layer thickness, so the worsened adhesion between the deposited filaments could be the reason for enhanced dimensional deviations. This conclusion is in accordance with the findings of previous research [52].

Regarding the effect of the filament color on the variation in the dimensional deviations with the layer height, the top views of the samples shown in Figure 4, Figure 5, Figure 6 and Figure 7 revealed the fact that, as above presumed, the same process parameters determined different printing conditions with respect to the state of the polymer, ranging from over-extrusion determined by too low viscosity of the filament (red PLA, 0.05 mm, Figure 4a) to under-extrusion caused by too high viscosity (black PLA, 0.20 mm, Figure 7d). Therefore, the reasons are most probably the differences regarding the glass transition temperatures and the thermal conductivities of different colored PLA filaments.

Furthermore, one can observe that the red PLA exhibited, in both directions (width and height), significantly larger dimensional deviations than the other colors. Whilst the surfaces of the red tensile specimens presented characteristic aspects for over-extrusion (non-uniform filament roads and oozing, marked in Figure 4), indicating a too high printing temperature, the increase in the layer thickness led to the appearance of air gaps between the filament roads, and, in the case of the 0.20 mm layer height, even to the delamination of successive layers. In accordance with previous research [10], a high printing temperature and thereby increased fluidity of the PLA filament allows the layer to expand in the side faces [10], leading to higher width deviations. On the other hand, the probably low thermal conductivity of the red PLA, and thereby its late response to the repetitive heating and cooling cycles, caused a high temperature gradient during the printing process with enhanced layer thickness. As also concluded by previous research [61], a high temperature gradient determines distortions within or between the layers, resulting in interlayer cracking.

### 3.2. Tensile Behavior

**Figure 8:**
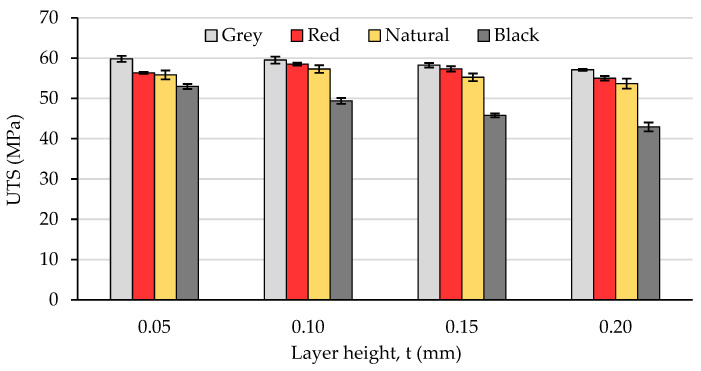
The variation in the ultimate tensile strength (UTS) with the layer height and the filament color.

The mean values of the UTS, calculated for the specimens manufactured with the same color-layer height combination as well as the standard deviations are presented in Figure 8.

The experimental results revealed beyond doubt that the tensile strength of the PLA prints is significantly color-dependent. As one can observe in Figure 8, the overall variation trend of the UTS vs. layer height was the same regardless of the PLA color, in the sense that the tensile strength decreases with increasing the layer height. On the other hand, the differences between the extreme values of the samples’ UTS, measured for different PLA colors, ranged between 4.77% (grey PLA) and 23.41% (black PLA), whilst the natural PLA and the red PLA manifested a very similar conduct (6.73% for natural PLA, 6.37% for the red PLA differences between the extreme UTS values obtained for different layer thicknesses). As also concluded by [13], the increased number of thinner layers intensified the re-heat effect for the already deposited layers and conducive to improved diffusion and adhesion between the layers. This phenomenon can be observed clearly in the mesostructure of the breakage surfaces of the samples, as presented in Figure 4, Figure 5, Figure 6 and Figure 7.

Furthermore, a slight increase in the UTS could be observed for the natural PLA printed with a 0.10 mm layer thickness and for the red PLA at 0.10 and 0.15 mm. This phenomenon may be explained by the fact that the re-heating cycles during the deposition process of the successive layers, created in the case of the two filament colors, the thermal conditions ensuring the maximum content of in-process crystallinity and thereby the superior mechanical strength of the samples, as also concluded in previous research [64].

Comparing the variation in the UTS vs. layer thickness (Figure 8) with that of the dimensional deviations vs. layer thickness (Figure 2 and Figure 3), two facts can be observed:

- The colors leading to better dimensional accuracy (respectively black and natural) unfortunately conduced to lower mechanical strength of the prints, caused by the weaker adhesion between the adjacent roads, as evidenced in the top-views of the tensile samples presented in Figure 6 and Figure 7. These bond deficiencies may be explained by the higher thermal conductivity of the natural PLA and black PLA in comparison to the grey and red filaments, and thereby by faster cooling rates of the filaments, with a subsequent faster contraction of the material, which led to the occurrence of adhesion loss between the deposited lines. Similar findings were also described by the authors of previous research [43,65];
- The effect of the layer thickness on the UTS values was more pronounced in the case of the black PLA, whose thermal conductivity was also reported by other researchers to be significantly higher than that of the other PLA filaments [66,67]. In this case, the difference between the UTS value for the sample printed with the 0.05 mm layer thickness and that realized with 0.20 mm amounted to 23.41%. This variation in the UTS values is in accordance with the mesostructure of the fractured surfaces presented in Figure 7, showing a continuous recrudescence of the bond between the deposited filaments with the layer thickness increase.

In order to find out whether the two independent variables, the PLA color and the layer height, had a significant influence on the ultimate tensile strength, a two way ANOVA test was performed under the following conditions:

- Nominal variable—the PLA color, expressed by four different colors (natural, black, grey, red);
- Ordinal variable—the layer height, expressed by four different thicknesses (0.05 mm, 0.10 mm, 0.15 mm, 0.20 mm);
- Dependent variable—the UTS, expressed in MPa.

The interaction effect of the two independent variables on the dependent variable was also evaluated.

The results of the two way ANOVA test rejected the three null hypotheses, thereby showing that the PLA color, the layer height, and the interaction between the PLA color and the layer height influence the tensile strength.

Moreover, the statistical analysis led to the conclusion that the strongest effect on the tensile strength is exerted by the PLA color (Partial Eta square η^2^ = 97.3%), followed by the layer height (η^2^ = 85.5%) and the interaction between the PLA color and the layer height (η^2^ = 80.0%). The confidence interval was set to 95%.

## 4. Conclusions

Aiming to point out if, and to what extent, the color of the PLA filament influenced the dimensional accuracy and the tensile strength of samples printed by FFF, the authors of the present research carried out experimental investigations on tensile ISO 527-2 type 1A specimens. The variable parameters were the layer height (0.05 mm, 0.1 mm, 0.15 mm, 0.20 mm) and the material color (natural, black, red, and grey). In order to avoid interactions of simultaneous varied parameters, all other process parameters were set at the same values for all samples as follows: printing temperature of 210 °C, build plate temperature of 60 °C, printing speed of 50 mm/s, 0.40 mm nozzle diameter, build orientation YX, raster angle 45°/−45°, infill density 100%, and two wall lines.

Based on the experimental results and the discussion presented in Chapter 3, the following conclusions can be drawn:

- First of all, the layer height influenced the dimensional accuracy of the PLA samples manufactured by FDM. While the trend of variation was the same regardless of the filament color, the experiments carried out within this study revealed that the coloring fillers determined significant differences with respect to the effective values of the dimensional deviations (7% up to 12% in the case of the cross-section area);
- The deviations of the samples’ dimensions differed depending on their positions in relation to the build orientation, as for all PLA colors, the thickness deviations of the samples exceeded the width deviations. The best dimensional accuracy was obtained for layer heights situated between 0.10 mm and 0.15 mm for all filament types. However, when considering the magnitude of the dimensional deviations, the influence of the PLA color was significant. Thus, for example, in the case of the thickness (*Z*-axis), the dimensional deviations of the samples printed with the 0.20 mm-layer height ranged between 5.48% (black PLA) and 12.19% (red PLA), whilst in the case of the width (*X*-axis), the values of the deviations were situated between 0.17% (black PLA) and 3.86% (red PLA) for the lowest layer height (0.05 mm);
- With respect to the tensile strength, the experimental results showed that regardless of the PLA color, the tensile strength decreased with the increment in the layer thickness. On the other hand, considering the magnitude of the variation in the UTS depending on the layer height for all of the investigated samples (4.77% for grey PLA up to 23.41% for black PLA), the results obtained within this study clearly reveal that the influence of the PLA color is also significant;
- Moreover, the two way ANOVA test performed by the authors in the case of the layer height—PLA filament color—UTS dependence revealed that the strongest effect on the tensile strength was exerted by the PLA color (partial Eta square η^2^ = 97.3%), followed by the layer height (η^2^ = 85.5%) and the interaction between the PLA color and the layer height (η^2^ = 80.0%);
- Finally, the experimental results showed that the colors leading to better dimensional accuracy (respectively black and natural) unfortunately led to the lower mechanical strength of the prints. Therefore, the color of the PLA filament has to be chosen depending on the targeted properties of the prints manufactured by FDM.

Considering the above, the overall conclusion that can be drawn is that any study regarding the optimization of the FDM process parameters for the 3D-printing of PLA, aiming to define the proper combinations of variables for the best quality and properties of the prints, should take into consideration the fact that coloring additives modify the thermal properties of the semi-crystalline PLA polymers and thereby their in-process behavior, so that, under the same printing conditions, different results in terms of the print’s quality and properties may be obtained. In other words, the color of the PLA filament used for the sample fabrication has to be mentioned among the process parameters as it is an influential factor that cannot be neglected.

## Figures and Tables

**Table 1:** The 3D-printing parameters.

Parameters		Values
Fixed process parameters	Printing head temperature, T_H_	210 °C
	Build plate temperature, T_B_	60 °C
	Printing speed, s_p_	50 mm/s
	Nozzle diameter, d_n_	0.40 mm
	Filament diameter, d_f_	2.85 mm
	Build orientation (acc. to [57])	YX
	Raster angle, θ	45°/−45°
	Infill density	100%
	Number of wall lines, W_L_ (-)	2
Variable parameters	Layer thickness, t	0.05 mm; 0.1 mm; 0.15 mm; 0.2 mm
	Material/Filament color	PLA Natural; PLA Black; PLA Red; PLA Grey

## Data Availability

The data presented in this study are available on request from the corresponding author.

## References

[1] Rouf S., Malik A., Singh N., Raina A., Naveed N., Siddiqui M.I.H., Haq M.I.U. (2022). Additive Manufacturing Technologies: Industrial and Medical Applications. Sustain. Oper. Comput..

[2] https://www.statista.com/topics/1969/additive-manufacturing-and-3d-printing/#topicOverview.

[3] (2021). Additive Manufacturing—General Principles—Terminology.

[4] Raquel G., Figueiredo-Pina C.G., Serro A.P. (2019). Additive manufacturing of ceramics for dental applications: A review. Dent. Mater..

[5] Rahmatabadi D., Ghasemi I., Baniassadi M., Abrinia K., Baghani M. (2022). 3D Printing of PLA-TPU with Different Component Ratios: Fracture Toughness, Mechanical Properties, and Morphology. J. Mater. Res. Technol..

[6] Aberoumand M., Soltanmohammadi K., Soleyman E., Rahmatabadi D., Ghasemi I., Baniassadi M., Abrinia K., Baghani M. (2022). A Comprehensive Experimental Investigation on 4D Printing of PET-G under Bending. J. Mater. Res. Technol..

[7] Schelly C., Anzalone G., Wijnen B., Pearce J.M. (2015). Open-source 3-D printing technologies for education: Bringing additive manufacturing to the classroom. J. Vis. Lang. Comput..

[8] Dey A., Yodo N. (2019). A Systematic Survey of FDM Process Parameter Optimization and Their Influence on Part Characteristics. J. Manuf. Mater. Process..

[9] Cuan-Urquizo E., Barocio E., Tejada-Ortigoza V., Pipes R.B., Rodriguez C.A., Roman-Flores A. (2019). Characterization of the mechanical properties of FFF structures and materials: A review on the experimental, computational and theoretical approaches. Materials.

[10] Valerga A.P., Batista M., Puyana R., Sambruno A., Wendt C., Marcos M. (2017). Preliminary study of PLA wire colour effects on geometric characteristics of parts manufactured by FDM. Procedia Manuf..

[11] Behzadnasab M., Yousefi A.A., Ebrahimibagha D., Nasiri F. (2020). Effects of processing conditions on mechanical properties of PLA printed parts. Rapid Prototyp. J..

[12] Kuznetsov V.E., Solonin A.N., Tavitov A., Urzhumtsev O., Vakulik A. (2020). Increasing strength of FFF three-dimensional printed parts by influencing on temperature-related parameters of the process. Rapid Prototyp. J..

[13] Cojocaru V., Frunzaverde D., Miclosina C.-O., Marginean G. (2022). The influence of the process parameters on the mechanical properties of PLA specimens produced by fused filament fabrication—A review. Polymers.

[14] Vanaei H., Shirinbayan M., Deligant M., Raissi K., Fitoussi J., Khelladi S., Tcharkhtchi A. (2020). Influence of process parameters on thermal and mechanical properties of polylactic acid fabricated by fused filament fabrication. Polym. Eng. Sci..

[15] Samykano M. (2021). Mechanical Property and Prediction Model for FDM-3D Printed Polylactic Acid (PLA). Arab. J. Sci. Eng..

[16] Lanzoti A., Grasso M., Staiano G., Martorelli M. (2015). The impact of process parameters on mechanical properties of parts fabricated in PLA with an open-source 3-D printer. Rapid Prototyp. J..

[17] Milovanovic A., Sedmak A., Grbovic A., Golubovic Z., Mladenovic G., Colic K., Milosevic M. Comparative analysis of printing parameters effect on mechanical properties of natural PLA and advanced PLA-X material. Proceedings of the 1st European-Structural-Integrity-Society (ESIS) Virtual European Conference on Fracture (ECF).

[18] Wang S.H., Ma Y.B., Deng Z.C., Zhang S., Cai J.X. (2020). Effects of fused deposition modeling process parameters on tensile, dynamic mechanical properties of 3D printed polylactic acid materials. Polym. Test.

[19] Yao T.Y., Deng Z.C., Zhang K., Li S.M. (2019). A method to predict the ultimate tensile strength of 3D printing polylactic acid (PLA) materials with different printing orientations. Compos. Part B Eng..

[20] Yao T.Y., Ye J., Deng Z.C., Zhang K., Ma Y.B., Ouyang H.J. (2020). Tensile failure strength and separation angle of FDM 3D printing PLA material: Experimental and theoretical analyses. Compos. Part B Eng..

[21] Yao T.Y., Zhang K., Deng Z.C., Ye J. (2020). A novel generalized stress invariant-based strength model for inter-layer failure of FFF 3D printing PLA material. Mater. Des..

[22] Zhao Y., Chen Y.S., Zhou Y.J. (2019). Novel mechanical models of tensile strength and elastic property of FDM AM PLA materials: Experimental and theoretical analyses. Mater. Des..

[23] Bayraktar O., Uzun G., Cakiroglu R., Guldas A. (2017). Experimental study on the 3D-printed plastic parts and predicting the mechanical properties using artificial neural networks. Polym. Adv. Technol..

[24] Altan M., Eryildiz M., Gumus B., Kahraman Y. (2018). Effects of process parameters on the quality of PLA products fabricated by fused deposition modeling (FDM): Surface roughness and tensile strength. Mater. Test..

[25] Laureto J.J., Pearce J.M. (2018). Anisotropic mechanical property variance between ASTM D638-14 type I and type IV fused filament fabricated specimens. Polym. Test.

[26] Hasan M.S., Ivanov T., Vorkapic M., Simonovic A., Daou D., Kovacevic A., Milovanovic A. (2020). Impact of Aging Effect and Heat Treatment on the Tensile Properties of PLA (Poly Lactic Acid) Printed Parts. Mater. Plast..

[27] Cardoso P.H.M., Teixeira B.N., Calado V.M.D., de Oliveira M.G., Mendonca T.D., Mendonca R.H., de Almeida H.R.O., Cunha M.S., Thire R. (2020). Mechanical and dimensional performance of poly(lactic acid) 3D-printed parts using thin plate spline interpolation. J. Appl. Polym. Sci..

[28] Lokesh N., Praveena B.A., Sudheer Reddy J., Vasu V.K., Vijaykumar S. (2021). Evaluation on Effect of Printing Process Parameter through Taguchi Approach on Mechanical Properties of 3D Printed PLA Specimens Using FDM at Constant Printing Temperature. Mater. Today Proc..

[29] Mazurchevici S.N., Pricop B., Istrate B., Mazurchevici A.D., Carlescu V., Carausu C., Nedelcu D. (2020). Technological Parameters Effects on Mechanical Properties of Biodegradable Materials Using FDM. Mater. Plast..

[30] Priya M.S., Naresh K., Jayaganthan R., Velmurugan R. (2019). A comparative study between in-house 3D printed and injection molded ABS and PLA polymers for low-frequency applications. Mater. Res. Express.

[31] Rajpurohit S.R., Dave H.K. (2018). Flexural strength of fused filament fabricated (FFF) PLA parts on an open-source 3D printer. Adv. Manuf..

[32] Rajpurohit S.R., Dave H.K. (2019). Analysis of tensile strength of a fused filament fabricated PLA part using an open-source 3D printer. Int. J. Adv. Manuf. Technol..

[33] Rajpurohit S.R., Dave H.K. (2021). Impact strength of 3D printed PLA using open source FFF-based 3D printer. Prog. Addit. Manuf..

[34] Rodriguez-Panes A., Claver J., Camacho A.M. (2018). The Influence of Manufacturing Parameters on the Mechanical Behaviour of PLA and ABS Pieces Manufactured by FDM: A Comparative Analysis. Materials.

[35] Alafaghani A., Qattawi A., Alrawi B., Guzman A. (2017). Experimental Optimization of Fused Deposition Modelling Processing Parameters: A Design-for-Manufacturing Approach. Procedia Manuf..

[36] Beniak J., Krizan P., Matus M. (2019). Mechanical properties of biodegradable pla plastic parts produced by 3D printing. MM Sci. J..

[37] Alafaghani A., Qattawi A. (2018). Investigating the effect of fused deposition modeling processing parameters using Taguchi design of experiment method. J. Manuf. Process..

[38] Bardiya S., Jerald J., Satheeshkumar V. (2021). The impact of process parameters on the tensile strength, flexural strength and the manufacturing time of fused filament fabricated (FFF) parts. Mater. Today Proc..

[39] Giri J., Chiwande A., Gupta Y., Mahatme C., Giri P. (2021). Effect of process parameters on mechanical properties of 3d printed samples using FDM process. Mater. Today Proc..

[40] Luzanin O., Movrin D., Stathopoulos V., Pandis P., Radusin T., Guduric V. (2019). Impact of processing parameters on tensile strength, in-process crystallinity and mesostructure in FDM-fabricated PLA specimens. Rapid Prototyp. J..

[41] Zisopol D.G., Nae I., Portoaca A.I., Ramadan I.A. (2021). Theoretical and Experimental Research on the Influence of FDM Parameters on Tensile Strength and Hardness of Parts Made of Polylactic Acid. Eng. Technol. Appl. Sci. Res..

[42] Frunzaverde D., Cojocaru V., Ciubotariu C.-R., Miclosina C.-O., Ardeljan D.D., Ignat E.F., Marginean G. (2022). The Influence of the Printing Temperature and the Filament Color on the Dimensional Accuracy, Tensile Strength, and Friction Performance of FFF-Printed PLA Specimens. Polymers.

[43] Gao G., Xu F., Xu J., Liu Z. (2022). Study of Material Color Influences on Mechanical Characteristics of Fused Deposition Modeling Parts. Materials.

[44] Pandžić A., Hodžić D., Milovanović A. Influence of Material Colour on Mechanical Properties of PLA Material in FDM Technology. Proceedings of the 30th DAAAM International Symposium on Intelligent Manufacturing and Automation.

[45] Spina R. (2019). Performance Analysis of Colored PLA Products with a Fused Filament Fabrication Process. Polymers.

[46] Marsavina L., Vălean C., Mărghitaş M., Linul E., Ali M., Berto F., Brighenti R. (2022). Effect of the Manufacturing Parameters on the Tensile and Fracture Properties of FDM 3D-Printed PLA Specimens. Eng. Fract. Mech..

[47] Al Rashid A., Abdul Qadir S., Koç M. (2021). Microscopic Analysis on Dimensional Capability of Fused Filament Fabrication Three-Dimensional Printing Process. J. Elastomers Plast..

[48] Mendricky R., Fris D. (2020). Analysis of the Accuracy and the Surface Roughness of FDM/FFF Technology and Optimisation of Process Parameters. Teh. Vjesn. Tech. Gaz..

[49] Biglete E.R., Dela Cuz J., Verdadero M.S., Manuel M.C., Altea A., Lubi A.J., Gatpayat A.G., Santos C.D. Dimensional Accuracy Evaluation of 3D—Printed Parts Using a 3D Scanning Surface Metrology Technique. Proceedings of the 11th IEEE Control and System Graduate Research Colloquium (ICSGRC).

[50] García Plaza E., Núñez López P., Caminero Torija M., Chacón Muñoz J. (2019). Analysis of PLA Geometric Properties Processed by FFF Additive Manufacturing: Effects of Process Parameters and Plate-Extruder Precision Motion. Polymers.

[51] Zharylkassyn B., Perveen A., Talamona D. (2020). Effect of Process Parameters and Materials on the Dimensional Accuracy of FDM Parts. Mater. Today Proc..

[52] Butt J., Bhaskar R., Mohaghegh V. (2022). Analysing the Effects of Layer Heights and Line Widths on FFF-Printed Thermoplastics. Int. J. Adv. Manuf. Technol..

[53] Milovanović A., Milošević M., Mladenović G., Likozar B., Čolić K., Mitrović N., Mitrovic N., Milosevic M., Mladenovic G. (2019). Experimental Dimensional Accuracy Analysis of Reformer Prototype Model Produced by FDM and SLA 3D Printing Technology BT—Experimental and Numerical Investigations in Materials Science and Engineering.

[54] Abas M., Habib T., Noor S., Salah B., Zimon D. (2022). Parametric Investigation and Optimization to Study the Effect of Process Parameters on the Dimensional Deviation of Fused Deposition Modeling of 3D Printed Parts. Polymers.

[55] Hanon M.M., Zsidai L., Ma Q. (2021). Accuracy Investigation of 3D Printed PLA with Various Process Parameters and Different Colors. Mater. Today Proc..

[56] (2012). Plastics—Determination of Tensile Properties—Part 2: Test Conditions for Molding and Extrusion Plastics.

[57] (2013). Standard Terminology for Additive Manufacturing—Coordinate Systems and Test Methodologies.

[58] (2019). Plastics—Determination of Tensile Properties—Part 1: General Principles.

[59] (1980). Statistical Interpretation of test Results–Estimation of the Mean—Confidence Interval.

[60] Soares J.B., Finamor J., Silva F.P., Roldo L., Cândido L.H. (2018). Analysis of the Influence of Polylactic Acid (PLA) Colour on FDM 3D Printing Temperature and Part Finishing. Rapid Prototyp. J..

[61] Li H., Wang T., Li Q., Yu Z., Wang N. (2018). A Quantitative Investigation of Distortion of Polylactic Acid/PLA) Part in FDM from the Point of Interface Residual Stress. Int. J. Adv. Manuf. Technol..

[62] Dave H.K., Prajapati A.R., Rajpurohit S.R., Patadiya N.H., Raval H.K. (2020). Investigation on Tensile Strength and Failure Modes of FDM Printed Part Using In-House Fabricated PLA Filament. Adv. Mater. Process. Technol..

[63] Taşcıoğlu E., Kıtay Ö., Keskin A.Ö., Kaynak Y. (2022). Effect of Printing Parameters and Post-Process on Surface Roughness and Dimensional Deviation of PLA Parts Fabricated by Extrusion-Based 3D Printing. J. Braz. Soc. Mech. Sci. Eng..

[64] Wittbrodt B., Pearce J.M. (2015). The effects of PLA color on material properties of 3-D printed components. Addit. Manuf..

[65] Ansari A.A., Kamil M. (2021). Effect of print speed and extrusion temperature on properties of 3D printed PLA using fused deposition modeling process. Mater. Today Proc..

[66] Beniak J., Šooš Ľ., Križan P., Matúš M., Ruprich V. (2022). Resistance and strength of conductive PLA processed by FDM additive manufacturing. Polymers.

[67] Zheng Y., Huang X., Chen J., Wu K., Wang J., Zhang X. (2021). A review of conductive carbon materials for 3D printing: Materials, technologies, properties, and applications. Materials.

